# Neonatal and Maternal 25-OH Vitamin D Serum Levels in Neonates with Early-Onset Sepsis

**DOI:** 10.3390/children4050037

**Published:** 2017-05-09

**Authors:** Taha Soliman Gamal, Abd-Allah Sayed Madiha, Mostafa Kamel Hanan, Mohamed El-Mazary Abdel-Azeem, Gamil S. Marian

**Affiliations:** 1 Pediatric Department, El-Minya University, Minya, 11432, Egypt; gamallt8@gmail.com (T.S.G.); Madiali445@gmail.com (A.-A.S.M.); maryyan999@gmail.com (G.S.M.); 2 Clinical-Pathology Department; El-Minya University, Minya 11432, Egypt; hanonamostafa62@gmail.com (M.K.H.)

**Keywords:** serum, 25-OH vitamin D, early onset, neonatal, sepsis

## Abstract

Vitamin D is a fat-soluble vitamin that is important for calcium metabolism and plays an important role in the immune functions. The aim of this study was to measure neonatal and maternal 25-OH vitamin D serum levels in neonates with early onset sepsis. The study included fifty neonates with early onset sepsis (25 full-term and 25 preterm infants) and thirty age and sex matched healthy neonates as controls. After history taking and clinical examination, complete blood count, C-reactive protein and 25-OH vitamin D serum levels (neonatal and maternal) were measured for all neonates. The mean gestational age for neonates with sepsis was (37.5 ± 0.98 for full term and 34.1 ± 1.26 for preterm neonates). Neonatal and maternal 25-OH vitamin D serum levels were significantly lower in patients (6.4 ± 1.8 and 24.6 ± 2.2 nmol/L) than controls (42.5 ± 20.7 and 50.4 ± 21.4 nmol/L). Significant negative correlations between neonatal and maternal 25-OH vitamin D serum levels and all sepsis markers and significant positive correlations between neonatal and maternal 25-OH vitamin D levels were present. At cut-off values <20 nmol/L for neonatal and <42 nmol/L for maternal 25-OH vitamin D for detection of neonatal sepsis, the sensitivity, specificity, positive predicted value (PPV) and negative predicted value (NPV) were 84%, 79%, 94.7% and 82.3% for neonatal and 82%, 77%, 91.4% and 80.6% for maternal 25-OH vitamin D, respectively. Positive correlations between neonatal and maternal 25-OH Vitamin D serum levels are present and they are negatively correlated with all sepsis markers. They can be sensitive early predictors for early onset sepsis in neonates.

## 1. Introduction

Neonatal sepsis is still a major cause of morbidity and mortality despite major advances in neonatal intensive care units. Clinical symptoms are generally subtle, but sepsis may rapidly progress and worsen, and may cause death within a few hours to days [1]. Many diagnostic biomarkers had been studied up to now, but none had achieved rapid and reliable enough identification specialty of infected neonates [2].

Early-onset sepsis (EOS) is an infection of the blood stream associated with a high morbidity and mortality. It is usually acquired vertically from the mother and manifests shortly after birth. In very low birth weight infants, the rates of mortality due to EOS are as high as 40%; therefore, an early recognition and initiation of antimicrobial therapy are of great importance in order to prevent morbidity and mortality [3]. Vitamin D, especially its active metabolite 1,25 di-hydroxy vitamin D3, plays an important role not only in calcium homeostasis and bone remodeling, but also in the control of hormone secretion, immune dysfunction, cell-proliferation and differentiation [4]. Recently, it has been well established that low levels of circulating 25-OH vitamin D have been shown to be strongly associated with infectious diseases. Although the mechanism of 25-OH Vitamin D on enhanced immunity is complex, it might have an important role in the optimal functions of the innate immune system by inducing antimicrobial peptides in epithelial cells, neutrophils and macrophages [5]. 

## 2. Patients and Methods

### 2.1. Patients

This is a prospective case-control study that included fifty neonates with early onset neonatal sepsis that occurred at <72 h. Criteria of neonatal sepsis included alteration in at least two of the following clinical signs: respiratory (distress, apnea, tachypnea, or hypoxemia), cardiological (tachycardia or bradycardia), hemodynamics (bad color, poor peripheral hypo perfusion, hypotension), neurological (irritability, lethargy, hypotonia, hypo activity, seizures), gastrointestinal (poor feeding, abdominal distension, feeding intolerance), temperature (fever > 38 °C, hypothermia < 36.0 °C), and metabolic (metabolic acidosis or hyperglycemia) plus elevated immature/ total neutrophils ratio (I/T ratio) > 0.2, presence of toxic neutrophil granules or elevated white blood cell count > 25,000 mm^3^ at the time of evaluation [6,7]. 

Maternal prenatal risk factors for EOS like premature rupture of membrane > 18 h, unclean foul, smelly turbid vaginal secretion, foul smelling liquor, maternal pyrexia with temp > 38 °C, maternal leukocytosis > 14,000, dysuria and prolonged labor more than 24 h were studied [8].

### 2.2. Controls

Thirty age and sex healthy neonates with no prenatal risk factor for EOS were enrolled in the study as a control group. 

All neonates were selected from the delivery room, El-Minya University hospital (Minya, Egypt) for children from March 2015 to May 2016. They were subjected to thorough history taking including: gestational age, age, sex, obstetric history (maternal temperature, duration of labor, amount and color of vaginal secretions), general examination including weight, vital signs, chest, heart, abdominal, neurological examination, umbilical stump status (infected or not) and neonatal activity (doing or not doing well). 

### 2.3. Laboratory Investigations 

For all neonates, two milliliters of venous blood were withdrawn under complete aseptic conditions for complete blood count (CBC), blood culture and C-reactive protein (CRP) within the first 72 h after birth. 25-OH vitamin D serum level was measured shortly after birth (within 24 h) by ELISA technique using Accubind ELISA micro wells (Monobind, Inc., Lakeforest, CA, USA) [9]. 

Another two milliliters of maternal blood were withdrawn within 24 h after birth for (25-OH vitamin D) assessment using a radioimmunoassay method in which we used Gamma-B 25-(OH)D RIA (IDS, Boldon, UK). The coefficient of variation (CV) for inter-assay analysis was 7.6% and sensitivity less than 3 nmol/L. All samples were analyzed in duplicate and all duplicates with a coefficient of variation more than 10% were reanalyzed [10]. 

25-OH vitamin D serum level values were categorized in descriptive analyses as sufficient with serum levels more than 50 nmol/L, insufficient between 30–50 nmol/L and deficient when <30 nmol/L according to institute of medicine (IOM) report and the consensus report on nutritional rickets [11,12,13].

The study was approved by the local research ethics committee of the El-Minya university hospital for children and written informed consents were obtained from the parents of all neonates to share in the study.

### 2.4. Statistical Analysis

The data were statistically analyzed using the SPSS software package, version 16 (SPSS Inc., Chicago, IL, USA) on a personal computer. Numerical data were expressed as mean ± standard deviation (SD). Non numerical data were expressed as frequencies. Comparative studies were done using a Student *t*-test and chi-square test. (*p*-value < 0.05 was considered significant). Correlation coefficients were used to describe associations between variables, and multiple regression analysis was used to detect any relationships between the variables. Receiver operating characteristic (ROC) curve analyses with measurements of area under the curve (AUC) were performed to identify the appropriate cut-off values.

## 3. Results

Fifty neonates with EOS were enrolled in this study. Twenty-five were full term who were 14 males (56%) and 11 females (44%). Seventeen of them (44%) delivered by caesarean section with mean birth weight 3.2 ± 0.43 kg and mean gestational age 37.5 ± 0.98 weeks. A history of maternal risk factors was found in 12 (48%) of them. Another 25 preterm neonates were enrolled who were 15 males (50%) and 15 (50%) females with main birth weight 2.78 ± 0.3 kg and mean gestational age 34.1 ± 1.26 weeks. Eighteen (72%) of them were delivered by caesarean section and 14 (56%) of them had a history of maternal risk factors for EOS (Table 1).

Hemoglobin level and platelets count were decreased in patients compared with controls (*p* < 0.001 for both) while white blood cells (WBCs), neutrophils counts, staff cells, segmented cells and I/T ratio all were significantly elevated in patients compared with controls (*p* < 0.001 for all) (Table 2).

Neonatal and maternal serum 25-OH vitamin D levels were more significantly decreased in preterm than in full term neonates in both groups (*p* = 0.02 and 0.01, respectively) and in all septicemic neonates compared with their corresponding controls (*p* < 0.001). 

In the patients group, 63% of women were vitamin-D deficient with vitamin-D serum levels less than 30 nmol/L and 20% were insufficient with levels between 30–50 nmol/L, while, in the control group, only 26% of women were 25-OH vitamin D deficient with 25-OH vitamin D serum levels less than 30 nmol/L, and 10% were insufficient with levels between 30–50 nmol/L. 

**Figure 1:**
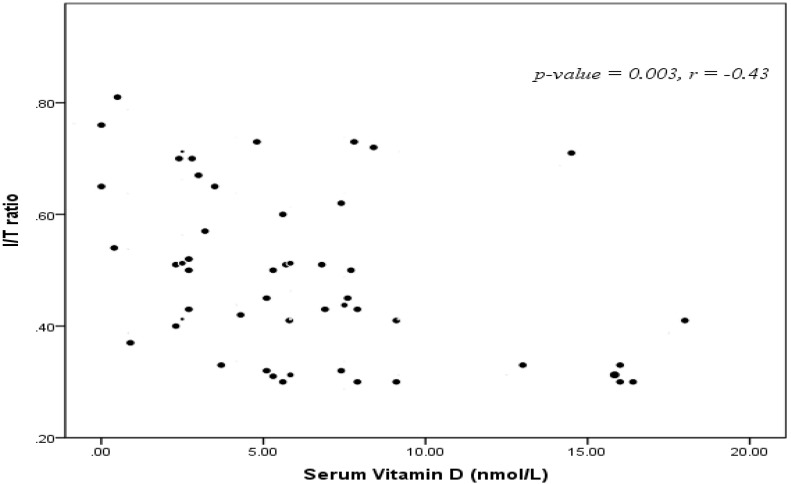
Correlation between 25-OH vitamin D serum level and immature/ total neutrophils ratio (I/T ratio).

**Figure 2:**
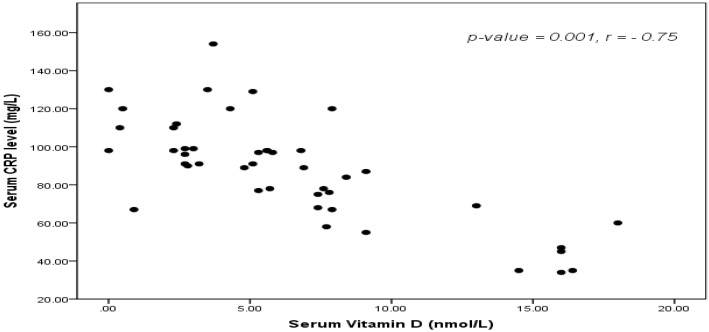
Correlation between 25-OH vitamin D serum level and C-reactive protein (CRP) levels.

Neonatal 25-OH vitamin D serum levels were positively correlated with maternal 25-OH vitamin D levels and negatively correlated I/T ratio (r = −0.43, *p* = 0.003) (Figure 1), interleukin-6 (IL-6) and CRP (r = –0.75, *p* < 0.001) (Figure 2). 

In this study, the cut-off values for predicting EOS was neonatal 25-OH vitamin D serum level <20 nmol/L. AUC 0.87 ± 0.12 (*p* = 0.004) with sensitivity 84%, specificity 79%, positive predictive value 94.7% and negative predictive value 82.3%. 

Also, significant negative correlations between maternal 25-OH Vitamin D levels and I/T ratio (r = –0.43, *p* = 0.003), CRP (r = −0.75, *p* < 0.001) and IL-6 were present. AUC 0.84 ± 0.23 (*p* = 0.02) with sensitivity 82%, specificity 77%, positive predictive value 91.4% and negative predictive value 80.6%. 

Single and multivariable logistic regression analysis with presence or absence of EOS as the dependent variable and 25-OH vitamin D with the many other measures as independent variables revealed that the most important factors affecting EOS were gestational age, IL-6 and neonatal 25-OH vitamin D followed by maternal 25-OH vitamin D, CRP level, maternal risk factors and the least factors were weight and gender.

## 4. Discussion 

In the present study, neonates with EOS had a significantly lower level of 25-OH D (preterm 5.5 ± 2.27 nmol/L), (full term 7.3 ± 6.18 nmol/L) when compared to healthy control groups of neonates (*p* < 0.001) agreeing with the results of Leo et al. [14], and there were strong significant negative correlations between 25-OH vitamin D and all sepsis markers like (I/T ratio, CRP and IL-6). This comes in agreement with many studies reported that 25-OH vitamin D insufficiencies were associated with higher sepsis severity [14,15,16]. 

The direction of this association between 25-OH vitamin D and EOS is open to debate. Is this lower level of 25-OH vitamin D a risk factor for sepsis due to its important immunologic role in pathophysiology of sepsis or is it a result of sepsis secondary to the acute inflammatory response to EOS as part of an acute phase reaction? We think that the maternal deficiency of 25-OH vitamin D as well as the strong correlations between maternal and neonatal 25-OH vitamin D suggest that this deficiency preceded the onset of sepsis and was not secondary to it. 

In accordance with our results, many studies reported that, during infancy, low concentrations of cord blood 25-OH vitamin D had been associated with increased incidence of sepsis in the first year of life [17] and available evidence suggested that 25-OH vitamin D deficiencies may be a predictor of sepsis and/or elevated mortality rate in critically ill patients, and its deficiency is strongly associated with the risk of blood culture positivity [18]. The proposed mechanism is that defects in macrophage functions and the production of pro-inflammatory cytokines may occur in 25-OH Vitamin D deficiency [17,18,19].

The vitamin D receptor (VDR) is widely expressed in all immune cellular subsets and VDR ligation by 25-OH vitamin D results in activation of key innate immune cells such as monocytes, macrophages, and neutrophils leading to enhanced chemotactic, phagocytic, and bactericidal activities on important innate immune cells [20,21] promotes the conversion of 25-OH vitamin D3 to the active form (1,25-OH vitamin D3) and induces production of anti-microbial peptides, such as cathelicidin, which inhibit the growth of Gram-positive and Gram-negative bacteria. The vitamin D/VDR complexes directly induce the expression of antimicrobial proteins such as β -defensin or cathelicidin in cells of the innate immune system [22].

In recent years, vitamin D was reported to have a complex effect on immune functions as it enhanced innate immunity while it also downregulated the acquired immune responses [4,5]. The mechanical barrier of the skin and other epithelial surfaces constitute the first barrier to infections and activated 25-OH vitamin D has an important role in maintaining the integrity of epithelial cells by encoding the proteins needed for several tight junctions [23]. 

In the present study, septic neonates had an elevated CRP level compared to controls. Measurement of CRP is considered to be of particular value as an indicator of bacterial infection [24]. However, the utility of CRP for the diagnosis of neonatal infection has been the subject of controversy because of its unsatisfactory sensitivity. The CRP concentration increases physiologically in newborns within the first days after birth. This dynamic behavior may in part account for the low diagnostic accuracy of CRP measurements in neonatal infection, particularly when measured shortly after birth [25]. 

Preterm neonates in this study had lower 25-OH vitamin D serum levels than full term neonates and this is in agreement with a previous report that concluded that preterm birth before 32 completed weeks of gestation is an independent risk factor for low 25-OH vitamin D levels at birth [26]. This could be explained in the light of the fact that preterm infants are at high risk for nutritional deficiencies and 25-OH vitamin D deficiency can be a part of global nutritional deficiency [8,27]. 

Many studies suggested that fetal and newborn concentrations of 25-OH D depend on and correlate with the maternal serum levels as the fetus has no endogenous production of 25-OH D and depends on transplacental transfer [28]. This occurs mainly in the third trimester, and, therefore, as we and others previously reported, preterm infants are at increased risk of 25-OH vitamin D deficiency [26,27,28], and this can explain the strong positive correlations between neonatal and maternal serum levels as well as the significant difference between mothers of septicemic neonates and controls as regards the state of serum 25-OH vitamin D levels [28]. On the other hand, 25-OH D deficiencies in newborns might be due to maternal deficiency and can be a predisposing factor rather than sequelae to early onset neonatal sepsis as we mentioned before. 

In our study, both of neonatal and maternal 25-OH vitamin D serum levels were good sensitive markers (84% and 82%, respectively) and good specific tests (79% and 77%, respectively) with positive predictive values (94.7% and 91.4%, respectively) and negative predictive values (82.3% and 80.6%, respectively) for early detection of neonatal sepsis, and these are in agreement with many previous studies [27,28].

In contrary to our results, Gokhanet et al. [29] reported that high serum 25-OH vitamin D levels are associated with pediatric sepsis, and this difference may be due to the age group and the different sample size. In addition, Ratzinger et al. [30] reported that 1,25-OH D but not 25-OH D can predict bacteraemia and both of them failed to predict sepsis and mortality in a prospective cohort study, but this difference between our results and Ratzinger’s may be attributed to the age group of patients, as all of Ratzinger’s patients were above 41 years old, the different types of infections recruited in his study (respiratory, gastrointestinal (GIT), urological and central nervous system (CNS) infections), and, lastly, the methodology and the time for assay of vitamin D levels.

In this study, maternal risk factors like premature rupture of membrane >18 h, unclean foul, smelly turbid vaginal secretion, foul smelling liquor, maternal pyrexia with temperature >38 °C, maternal leukocytosis >14,000 were significantly higher in patients group than controls, and this can reflect the importance of maternal 25-OH vitamin D on the immune functions of the mothers themselves as well as of their offspring.

El-Mazary et al. [31] reported that there were positive correlations between the maternal serum levels of 25-OH vitamin D during the last trimester and with the neonatal levels of 25-OH vitamin D as well as with the incidence of infections in the neonatal period, and these results support the results of our study.

In this study, the interpretation of 25-OH vitamin D serum levels according to the recently published reports [11,12,13] revealed that, in this study, 63% of women in the patients group were vitamin-D deficient and 20% were insufficient, while, in controls, 26% of women were vitamin-D deficient and 10% were insufficient, which means that there were about 45% of the total women who were 25-OH vitamin D deficient, and 15% were insufficient in spite of our sunny environment. This high percentage of women suffering from 25-OH vitamin D deficiency may be due to increased time spent indoors, skin coverage with clothing and bad dietetic habits decreasing 25-OH vitamin D absorption, a problem that needs to be studied and solved with supplementation of 25-OH vitamin D during pregnancy [31,32].

Vitamin D deficiency is a worldwide problem [11] and many studies reported similar rates of vitamin D deficiency in Europe, Canada and Middle Eastern countries [33,34,35]. 

A study from the Middle East showed that 72.8% of adult population had 25(OH)D deficiency with higher incidence in women than in men (83.9% vs 48.5%, respectively) [35] and, in Boston, a study reported that vitamin D insufficiency was present in about two thirds of healthy, young adults [36]. 

In the present study, multiple regression analysis with presence or absence of EOS as the dependent variable and 25-OH D with the many other measures as independent variables revealed that the most important factors affecting EOS were gestational age, IL-6 and neonatal 25-OHD, supporting the strong relationship between EOS and the previous parameters as mentioned before, especially gestational age, which affects all other sepsis markers such as IL-6 and 25-OH vitamin D [26,27,31].

There were many limitations that had to be disclosed in this study. We measured maternal serum 25-OH vitamin D levels only at birth with no data regarding its serum levels during different trimesters, and we could not measure vitamin D binding protein (VDP), which is essential for suggesting that the lower levels of 25-OH vitamin D were primary and not secondary to sepsis. In addition, the sample size of this study was small and we did not have enough data about the causative organisms of EOS and their relation to 25-OH vitamin D. 

## 5. Conclusions

Positive correlations between neonatal and maternal 25-OH vitamin D serum levels are present and they are negatively correlated with all sepsis markers. They can be sensitive early predictors for early onset sepsis in neonates. 25-OH vitamin D supplementation for pregnant women may be of significant value in the future, and this point needs more research work.

## Figures and Tables

**Table 1:** Demographic data of studied neonates.

Item		Full-term (*n* = 40)			Preterm (*n* = 40)		
		Patients (*n* = 25)	Control (*n* = 15)	*p*-value	Patients (*n* = 25)	Control (*n* = 15)	*p*-value
Gestational age (weeks)	Mean ± SD	37.5 ± 0.98	37.4 ± 0.58	0.08	34.1 ± 1.26	35.2 ± 2.14	0.15
Birth weight (kg)	Mean ± SD	3.2 ± 0.43	3.19 ± 0.35	0.08	2.78 ± 0.3	2.14 ± 0.26	0.06
Gender	Male Female	14 (56%) 11 (44%)	12 (80%) 3 (20%)	0.19	15 (60%) 10 (40%)	8 (53%) 7 (47%)	0.63
Mode of delivery	NVD C.S	17 (68%) 8 (32%)	12 (80%) 3 (20%)	0.54	7 (28%) 18 (72%)	3 (20%) 12 (80%)	0.66
Maternal risk factors	Yes No	13 (52%) 12 (48%)	3 (20%) 12 (80%)	0.04	14 (56%) 11 (44%)	13 (86.6%) 2 (13.4%)	0.04

SD: standard deviation; NVD: normal vaginal delivery; CS: caesarean section.

**Table 2:** Some laboratory data of studied neonates.

Item	Patients (*n* = 50)	Controls (*n* = 30)	*p*-Value
Hb (gm/dL)	12.9 ± 3.4	15.4 ± 2.2	0.04
WBCs (×10³/µL)	20,038 ± 18,237.4	10,304 ± 3201.6	0.003
Platelets (×10³/µL)	89 ± 8.4	255 ± 5.9	0.002
Neutrophils(×10³/µL)	63.2 ± 12.9	37.5 ± 7.7	0.001
Staff (×10³/µL)	11.8 ± 7.4	3.0 ± 1.2	0.004
Segmented(×10³/µL)	51.9 ± 11.9	34.4 ± 7.5	0.01
ANC	11,184.5 ± 75.7	3847.2 ± 13.7	0.001
I/T ratio	0.2 ± 0.1	0.1 ± 0.03	0.01
CRP (mg/L)	37.6 ± 9.02	6.0 ± 1.05	0.001
IL-6 (pg/mL)	139.9 ± 70.19	5.8 ± 3.8	0.001
Neonatal. 25-OH Vit.D (nmol/L)	6.4 ± 1.8	24.6 ± 2.2	0.001
Maternal. 25-OH Vit.D (nmol/L)	42.5 ± 20.7	50.4 ± 21.4	0.01

Hb: hemoglobin; WBCs: white blood cells; ANC: Absolute Neutrophil Count; I/T ratio: immature/total neutrophils ratio; CRP: C-reactive protein; IL-6: interleukin 6.

## References

[1] Satar M., Ozlü J. (2012). Neonatal sepsis: A continuing disease burden. Pediatr..

[2] Dollner H., Vatten L., Austgalen R. (2001). Early diagnostic markers for neonatal sepsis: Comparing C-reactive protein, interleukin-6, soluble tumor necrosis factor receptors and soluble adhesion molecules. J. Clin. Epidemiol..

[3] Cizmeci M.N., Kara S., Kanburoglu M.K., Simavli S., Duvan C.I., Tatli M.M. (2014). Detection of cord blood hepcidin levels as a biomarker for early-onset neonatal sepsis. Med. Hypotheses.

[4] Di Rosa M., Malaguarnera M., Nicoletti F., Malaguarnera L. (2011). 25 OH Vitamin D3: A helpful immuno-modulator. Immunology.

[5] Kempker J.A., Han J.E., Tangpricha V., Ziegler T.R., Martin G.S. (2012). 25-OH Vitamin D and sepsis: An emerging relationship. Dermato-endocrinol..

[6] Stefanovic I.M. (2011). Neonatal sepsis. Biochemia. Medica..

[7] Tappero E., Johnson P. (2010). Laboratory Evaluation of Neonatal Sepsis. Newborn Infant Nurs. Rev..

[8] Burris H.H., Van Marter L.J., McElrath T.F., Tabatabai P., Litonjua A.A., Weiss S.T., Christou H. (2014). Vitamin D status among preterm and full-term infants at birth. Pediatr. Res..

[9] Herrmann M., Farrell C.L., Pusceddu I., Fabregat-Cabello N., Cavalier E. (2017). Assessment of vitamin D status — a changing landscape. Clin. Chem. Lab. Med..

[10] Snellman G., Melhus H., Gedeborg R., Byberg L., Berglund L., Wernroth L., Michaëlsson K. (2010). Determining Vitamin D Status: A Comparison between Commercially Available Assays. PLoS ONE.

[11] Ross A.C., Manson J.E., Abrams S.A., Aloia J.F., Brannon P.M., Clinton S.K., Durazo-Arvizu R.A., Gallagher J.C., Gallo R.L., Jones G. (2011). The 2011 Report on Dietary Reference Intakes for Calcium and Vitamin D from the Institute of Medicine: What clinicians need to know. J. Clin. Endocrinol. Metab..

[12] Manson J.E., Brannon P.M., Rosen C.J., Taylor C.L. (2016). Vitamin D Deficiency — Is There Really a Pandemic?. N. Engl. J. Med..

[13] Munns C.F., Shaw N., Kiely M., Specker B.L., Thacher T.D., Ozono K., Michigami T., Tiosano D., Mughal M.Z., Mäkitie O. (2016). Global Consensus Recommendations on Prevention and Management of Nutritional Rickets. J. Clin. Endocrinol. Metab..

[14] Jeng L., Judd S.E., Blumberg H.M., Martin G.S., Ziegler T.R., Tangpricha V. (2009). Alterations in 25-OH Vitamin D status and anti-microbial peptide levels in patients in the intensive care unit with sepsis. J. Transl. Med..

[15] Rech M.A., Hunsaker T., Rodriguez J. (2014). Deficiency in 25-hydroxy Vitamin D and 30-day mortality in patients with severe sepsis and septic shock. Am. J. Crit. Care..

[16] Amrein K., Litonjua A.A., Moromizato T., Quraishi S.A., Gibbons F.K., Pieber T.R., Camargo C.A., Giovannucci E., Christopher K.B. (2016). Increases in pre-hospitalization serum 25(OH) D concentrations are associated with improved 30-day mortality after hospital admission: A cohort study. Clin. Nutr..

[17] Ginde A.A., Camargo C.A.J., Shapiro N.I. (2011). 25-OH Vitamin D insufficiency and sepsis severity in emergency department patients with suspected infection. Acad. Emerg. Med..

[18] Shin Y.H., Yu J., Kim K.W., Ahn K., Hong S.A., Lee E., Yang S.I., Jung Y.H., Kim H.Y., Seo J.H. (2013). Association between cord blood 25-hydroxy Vitamin D concentrations and respiratory tract infections in the first 6 months of age in a Korean population: a birth cohort study (COCOA). Korean J. Pediatr..

[19] Braun A., Chang. D., Mahadevappa K., Gibbons F.K., Liu Y., Giovannucci E., Christopher K.B. (2011). Association of low serum 25-hydroxy Vitamin D levels and mortality in the critically ill. Crit. Care Med..

[20] Izban M.G., Nowicki B.J., Nowicki S. (2012). 1,25-dihydroxy Vitamin D3 promotes a sustained LPS-induced NF-κB-dependent expression of CD55 in human monocytic THP-1 cells. PLoS ONE.

[21] Liu P.T., Stenger S., Li H., Wenzel L., Tan B.H., Krutzik S.R., Ochoa M.T., Schauber J., Wu K., Meinken C. (2006). Toll-like receptor triggering of a 25 OH Vitamin D-mediated human antimicrobial response. Science.

[22] Gombart A.F., Borregaard N., Koeffler H.P. (2005). Human cathelicidin antimicrobial peptide (CAMP) gene is a direct target of the 25-OH Vitamin D receptor and is strongly up-regulated in myeloid cells by 1,25-dihydroxy Vitamin D3. FASEB J..

[23] Watkins R.R., Lemonovich T.L., Salata R.A. (2015). An update on the association of Vitamin D deficiency with common infectious diseases. Can. J. Physiol. Pharmacol..

[24] Chiesa C., Natale F., Pascone R. (2011). C-reactive protein and procalcitonin: reference intervals for preterm and term newborns during the early neonatal period. Clin. Chim. Acta.

[25] Posen R., Delemos R.A. (1998). C-reactive protein levels in the extremely premature infant: Case studies and literature review. J. Perinatol..

[26] Shah M.D., Shah S.R. (2009). Nutrient deficiencies in the premature infant. Pediatric Clin. North. Am..

[27] McCarthy R.A., McKenna M.J., Oyefeso O., Uduma O., Murray B.F., Brady J.J., Kilbane M.T., Murphy J.F., Twomey A., Donnell C.P. (2013). Vitamin D nutritional status in preterm infants and response to supplementation. Br. J. Nutr..

[28] Weisman Y. (2003). Maternal, fetal and neonatal 25-OH Vitamin D and calcium metabolism during pregnancy and lactation. Endocr. Dev..

[29] Aydemir G., Cekmez F., Kalkan G., Fidanci M.K., Kaya G., Karaoglu A., Meral C., Arzıman İ., Karademir F., Ayar G. (2014). High Serum 25-Hydroxyvitamin D Levels Are Associated with Pediatric Sepsis. Tohoku J. Exp. Med..

[30] Ratzinger F., Haslacher H., Stadlberger M., Schmidt R.L.J., Obermüller M., Schmetterer K.G., Perkmann T., Makristathis A., Marculescu R., Burgmann H. (2017). 25(OH)D and 1,25(OH)D vitamin D fails to predict sepsis and mortality in a prospective cohort study. Sci. Rep..

[31] El-Mazary A.M., Abdel-Maaboud M., Mohamed M., Nasef K.A. (2012). 25-OH Vitamin D supplementation and the risk of infections in fullterm infants. Correlations with the maternal serum 25 OH Vitamin D.. Arch. Dis. Child..

[32] Binkley N., Novotny R., Krueger D., Kawahara T., Daida Y.G., Lensmeyer G., Hollis B.W., Drezner M.K. (2007). Low Vitamin D status despite abundant sun exposure. J. Clin. Endocrinol. Metab..

[33] de Haan K., Groeneveld A.J., de Geus H.R., Egal M., Struijs A. (2014). Vitamin D deficiency as a risk factor for infection, sepsis and mortality in the critically ill: Systematic review and meta-analysis. Crit. Care..

[34] Wang T.J., Zhang F., Richards J.B., Kestenbaum B., van Meurs J.B., Berry D., Kiel D.P., Streeten E.A., Ohlsson C., Koller D.L. (2010). Common genetic determinants of vitamin D insufficiency: A genome-wide association study. Lancet.

[35] Berry D.J., Vimaleswaran K.S., Whittaker J.C., Hingorani A.D., Hypponen E. (2012). Evaluation of Genetic Markers as Instruments for Mendelian Randomization Studies on Vitamin D. PloS ONE.

[36] Barragan M., Good M., Kolls J.K. (2015). Regulation of Dendritic Cell Function by Vitamin D. Nutrients.

